# A Calcium-Dependent Mechanism of Neuronal Memory

**DOI:** 10.1371/journal.pbio.1002182

**Published:** 2015-06-22

**Authors:** Gabriel Gasque

**Affiliations:** Public Library of Science, San Francisco, California, United States of America

## Abstract

A neuron's record of its previous activity underlies animal memory. A new study reveals a role for the release of calcium ions from intracellular stores in mediating spatially compartmentalized memory of the activity history of a neuron.

Every fact or task that we remember—the shape of the utensil we call a fork, the appropriate hand-motion needed to beat egg whites until fluffy, or the sequential steps involved in baking a cake—must be encoded by long-lasting changes in the way that our neurons function and in the strength with which they connect and communicate to each other. Current experimental evidence has led neuroscientists to propose that learning elicits a particular pattern of electrical activity in neurons, which can in turn induce changes in their morphology, their responsiveness to incoming signals, the expression of their genes, and the strength of their connection to other neurons.

These changes are the cellular counterpart of what we think of as memory. However, neuroscientists have not found many mechanisms by which a neuron can store information relative to its previous activity. In a study just published in *PLOS Biology*, Friedrich Johenning, Anne-Kathrin Theis, Dietmar Schmitz, Sten Rüdiger, and colleagues provide evidence that specific electrical activity within neurons induces a long-lasting change in the amplitude of transitory increases of calcium ion concentration (Ca^2+^ transients) inside dendritic spines—the specialized protrusions of the dendrites of a neuron, which receive input from other neurons via synapses.

The Ca^2+^ ion is a fundamental player in the transformation of electrical to biochemical activity within neurons. The concentration of Ca^2+^ ions within a neuron can be increased either by influx from the extracellular milieu via ion channels and transporters or by release from intracellular stores via Ca^2+^-permeable channels like the ryanodine receptor (RyR). Once free in the cytosol, Ca^2+^ can bind to and change the activity of many proteins, including those that control gene expression, cell morphology, and the activity of other proteins via the addition or removal of phosphate groups. Because Ca^2+^ can have such broad impacts on the cell’s biochemistry, to ensure specificity of effect, the diffusion of the Ca^2+^ signal can be restricted to compartments, such as those enclosed by dendritic spines.

When a neuron gets activated, it fires an action potential, a short-lasting change in membrane potential that travels unidirectionally from near the cell body to the axon terminal, where it triggers the release of a chemical signal—a neurotransmitter—into the synaptic cleft, to be sensed by the adjacent neuron. However, action potentials also propagate back to the cell body and the dendrites of neurons. Johenning, Theis, and colleagues demonstrated that back-propagating action potentials are not only coupled to the activation of voltage-gated Ca^2+^ channels in the plasma membrane of the dendritic spine but also to the release of Ca^2+^ from intracellular stores via ryanodine receptors. More importantly, their study shows that when a dendritic spine sees a short burst of action potentials—similar to patterns of activity seen in freely moving animals—the Ca^2+^ transients elicited by subsequent back-propagating action potentials are enhanced for several minutes after the burst. These results indicate that the neuron can “remember” its previous history of activity and express this memory as a sustained increase in the amplitude of Ca^2+^ transients in a spine-specific manner (Fig 1).

**Fig 1:**
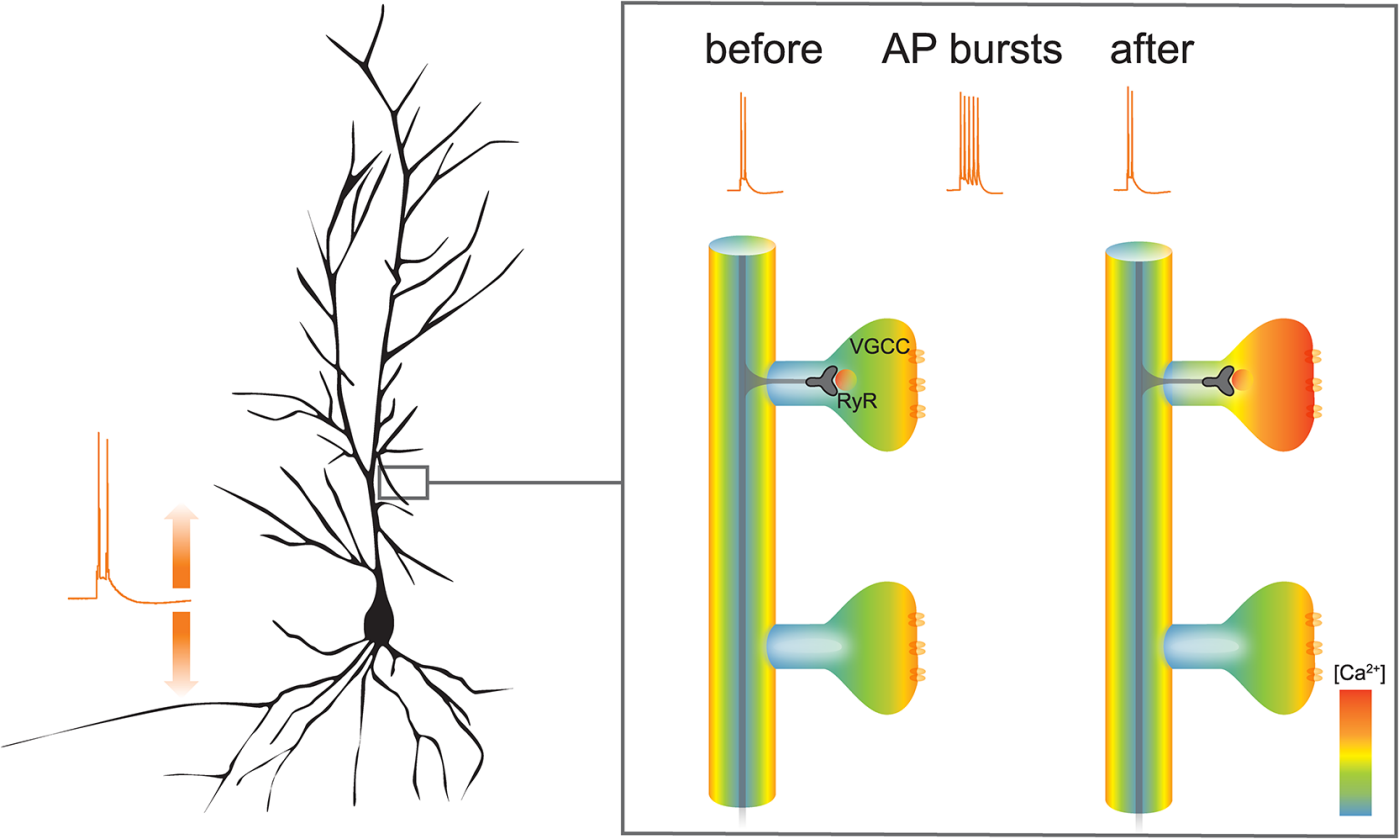
A new mechanism of storing patterns of previous neuronal activity: back-propagating action potentials activate voltage-gated Ca^2+^ channels (VGCCs) and ryanodine receptor (RyR) intracellular Ca^2+^ release channels; spine-specific Ca^2+^ memory is the result of Ca^2+^ release from intracellular stores via ryanodine receptors. AP, action potential. *Image credit*: *Friedrich Johenning*, *Ulrike Pannasch*, *and Dietmar Schmitz*.

The authors performed their experiments in neurons from different regions of the rat brain implicated in memory storage. The contribution of ryanodine receptors to the Ca^2+^ transient enhancement was evaluated using different pharmacological approaches—drugs that emptied the Ca^2+^ stores or blocked the ryanodine receptors also eliminated the enhancement, but only marginally affected the transients themselves, which were mostly mediated by voltage-gated Ca^2+^ channels.

Intriguingly, by measuring Ca^2+^ concentration variations on several spines simultaneously, the authors discovered that enhancement of Ca^2+^ transients was compartmentalized; it happened in a spine-restricted manner, without diffusing from one spine into another.

Because the spines are specialized in receiving incoming information from other neurons, it was a natural question for the authors to ask whether synaptic activity could be contributing to the enhancement of Ca^2+^ transients. Surprisingly, a cocktail of pharmacological agents that blocked the main types of excitatory and inhibitory transmission in the brain did not block the sustained increase in Ca^2+^ transients induced by back-propagating action potentials. The cytoplasmic Ca^2+^ elevation induced by the activation of the ryanodine receptors is therefore the most likely biochemical link between the activity-dependent membrane depolarization (i.e., the initial back-propagating action potentials) and the downstream signaling events that result in Ca^2+^ transient enhancement. This idea was supported by the fact that if a Ca^2+^ buffer was used to blunt the cytosolic Ca^2+^ increase during the train of back-propagating action potentials then the enhancement was eliminated.

The authors discovered that, surprisingly, emptying the intracellular Ca^2+^ stores after the burst of back-propagating action potentials did not affect potentiation. This result indicates that release of Ca^2+^ from the stores via ryanodine receptors is important for the initial establishment, but not for the subsequent expression, of the enhancement of Ca^2+^ transients.

Because it is impossible to experimentally measure the temporal and spatial distribution of Ca^2+^ ions in the tiny volume of the dendritic spines, the author used mathematical modeling to determine that the Ca^2+^ released via the ryanodine receptors acted on a (still unknown) target in a specific nanodomain close to the intracellular stores during the initial establishment of the Ca^2+^ transient enhancement induced by back-propagating action potentials.

Many questions remain open. For example, it is not clear why the back-propagating action potentials induce Ca^2+^ transient potentiation enhancement in only about half of the spines studied. In addition, the physiological stimuli that induce this effect—and the consequences of the sustained Ca^2+^ increases on the morphology and function of the dendritic spines—were not investigated. In the end, exactly how this potentiation modulates the way in which neurons communicate with each other is still unknown. This research by Johenning and colleagues is an important step forward, since it uncovers a new spine-restricted mechanism of storing the patterns of previous neuronal activity and defines the role of intracellular Ca^2+^ stores and ryanodine receptors in this form of cellular memory.
